# Blood routine reference value range should be adjusted according to regional and ethnic characteristics

**DOI:** 10.3389/fpubh.2022.934101

**Published:** 2022-07-29

**Authors:** Yan Guo, Xiao Liu, Zhang Zihao, Qiang Zhang, Zhongshan Shi, Na Zhang

**Affiliations:** ^1^Graduate school, Medical College of Soochow University, Suzhou, China; ^2^Department of Pathology, Qinghai Provincial People's Hospital, Xining, China; ^3^Department of Basic Medical Sciences, The 960th Hospital of PLA, Jinan, China; ^4^Department of Experimental Medicine, Hainan Hospital of PLA General Hospital, Sanya, China; ^5^Department of Neurosurgery, Qinghai Provincial People's Hospital, Xining, China; ^6^Department of Intensive Care Medicine, Ge er mu People's Hospital, Ge er mu, China; ^7^Department of Respiratory, Qinghai Provincial People's Hospital, Xining, China

**Keywords:** routine blood test, region, nationality, Li nationality, Tibetan

## Abstract

**Objective:**

To further understand the influence of regional and ethnic factors on blood routine indicators.

**Methods:**

The routine blood test (RBT) results of 617 healthy men aged 18–45 years old of the Li, Tibet, and Han nationalities living in the city of Sanya, Hainan Province (200 m), the city of Xining, Qinghai Province (2,300 m), and Maduo County of Qinghai Province (4,300 m) for a long time were studied. Eight indexes, such as the red blood cell (RBC), hemoglobin (Hb), and platelet (PLT) counts, were compared and analyzed.

**Results:**

With an increase in altitude, the RBT index values and change trends of the different ethnic groups were different. When the altitude increased by 2,000 m, the RBC and Hb increased by 6.6 and 8.1%, respectively, and the PLT decreased by 16.8%. However, the RBC, Hb, and PLT of the Tibetan subjects decreased by 7.4, 5.1, and 3.0%, respectively. In the same region, there were also significant differences in the RBT index values among the ethnic groups. The RBC increased, Hb decreased, and PLT did not change in the Li nationality in Sanya compared with the Han nationality. The RBC, Hb, and PLT of Tibetans in the Xining area were significantly higher than those of the Han population. Referring to the current RBT reference value range in China, the abnormal rates of the various RBT index values of the enrolled population were high. By utilizing Hb as an example, 27.7% of the Li nationality in Sanya was low, 67.0% of the Tibetan nationality in Xining was high, and 89.4% of the Maduo Han nationality was high. The PLT was lower in the Sanya Li nationality (13.8%) and the Maduo Han nationality (88.3%).

**Conclusion:**

Regional and ethnic factors have a significant impact on the RBT, and the current range of normal values of the RBT in China needs to be revised and adjusted.

## Introduction

A routine blood test (RBT) is the most common test item in the clinic. China has 56 ethnic minorities, and the altitude difference between the East and the West is >5,000 m. However, the current reference values for the RBT are divided only according to age and gender, and divisions of regions and ethnicities have not been introduced. Fully understanding the RBT values of various ethnicities is of great significance for disease diagnosis and prevention for patients in different regions and ethnic groups. Therefore, this study enrolled healthy men of the Li, Han, and Tibetan nationality aged 18–45 who had lived in the city of Sanya, Hainan Province (with an average altitude of 200 m), the city of Xining, Qinghai Province (2,300 m), and Maduo County (4,300 m) for a long time. Their red blood cell count (RBC), hematocrit (HCT), hemoglobin content (HB), mean red blood cell volume (MCV), mean red blood cell hemoglobin (MCH), mean red blood cell hemoglobin concentration (MCHC), platelet count (PLT), and platelet specific volume (PCT) were compared and analyzed to further understand the influence of regional and ethnic factors.

## Research object and method

### Selection of research objects

This study was approved by the ethics committee of the Hainan Hospital of the General Hospital of the Chinese People's Liberation Army (PLA) and the Qinghai Provincial People's Hospital. From November to December 2021, people who met the following standards underwent RBT in the above two hospitals, and their RBT result data was utilized as the research object.

#### Inclusion criteria

(1) Male, 18–45 years old;
(2) Li, Tibetan, or Han nationality;
(3) Long-term residence in Sanya, Xining, or Maduo for more than 3 years;
(4) Had not left his residence within half a year prior to the RBT;
(5) Conscious of no physical discomfort;
(6) Complete records of personal information and RBT results.

#### Exclusion criteria

Clinically diagnosed with a malignant tumor, cor pulmonale, hepatitis, nephritis, immune disease, serious infection, and other diseases that may affect the results of the RBT.

### Sample size calculation

The sample size estimation method (1) is described in the literature, and the different regions are reported in the literature (Shanghai and Xining) (2) (Table 1). In addition, different nationalities (Tibetan and Han) (3) (Table 2) and the minimum sample size required for this study were estimated. When using α = 0.05 and β = 0.10, the μ value table was produced and μ_α_ = 1.96 (bilateral) and μ_β_ = 1.28 (one side). This produced μ_α_, μ_β_, σ, and δ. The values were substituted into the equation [*n* = 2 (μ_α_ + μ_β_)^∧^2σ^∧^2/δ^∧^2] to calculate the minimum sample size. Then, according to the calculation results and combined with the actual situation, the minimum sample size of this study was determined.

**Table 1:** Regional factors and minimum sample size.

**Index**	**Region1 (Shanghai)**		**Region2 (Xinning)**		**δ**	**σ**	***n***
	$\bar{x}$ **1**	**s1**	$\bar{x}$ **2**	**s2**			
RBC	5.21	0.33	5.63	0.41	0.4	0.4	16.5
HB	156	8	172	11	16.0	9.6	7.6
MCV	88.1	3.4	89.7	3.7	1.6	3.6	103.6

*$\bar{x}$, mean value; s, standard deviation; μα = 1.96, μβ = 1.28, δ=|$\bar{x}$1 – $\bar{x}$2|, σ = [(s1^∧^2+s2^∧^2)/2]^∧^1/2, n = 2 (μα + μβ)^∧^2σ^∧^2/δ^∧^2. RBC, red blood cell count; HB, hemoglobin content; MCV, mean red blood cell volume*.

**Table 2:** Ethnic factors and minimum sample size.

**Index**	**Ethnic1 (Tibetan)**		**Ethnic2 (Han)**		**δ**	**σ**	***n***
	$\bar{x}$ **1**	**s1**	$\bar{x}$ **2**	**s2**			
RBC	5.35	0.45	6.04	0.50	0.7	0.5	10.0
HB	194.11	15.01	201.73	16.30	7.6	15.7	88.8
MCV	100.54	10.32	94.73	3.70	5.8	7.8	37.4
MCH	36.85	5.63	33.42	2.13	3.4	4.3	32.3
MCHC	361.79	31.14	352.19	13.75	9.6	24.1	132.0

*$\bar{x}$, mean value; s, standard deviation, μ_α_ = 1.96, μ_β_ = 1.28, δ = |$\bar{x}$1 – $\bar{x}$2|, σ = [(s1^∧^2 + s2^∧^2)/2]^∧^1/2, n = 2(μ_α_ + μ_β_)^∧^2σ^∧^2/δ^∧^2. RBC, red blood cell count; HB, hemoglobin content; MCV, mean red blood cell volume; MCH, mean red blood cell hemoglobin; MCHC, mean red blood cell hemoglobin concentration*.

### Elimination of deviation

The RBT results are affected by the living environment, gender, age, and health status. We strictly limited the entry conditions to men aged 18–45 who have lived in Sanya, Xining, and Maduo for more than 3 years and have not traveled to different places within half a year. They also had no conscious physical discomfort. After entry, we further excluded those diagnosed with malignant tumors, blood diseases, hepatitis, nephritis, serious infections, and other diseases so as to avoid deviation in the results.

The RBT results collected in this study were obtained from three laboratories (the Sanya data were collected from the laboratory department of Hainan Hospital of the PLA General Hospital, the Xining data were collected from the laboratory department of the Qinghai Provincial People's Hospital, and the Maduo County data were collected from the Maduo County outpatient department of the Qinghai Provincial People's Hospital). Owing to the different instruments, reagents, testers, and testing environments utilized for the RBT in the different laboratories, prior to the formal test, 10 peripheral blood samples that remained after clinical testing were collected in the three laboratories of the hospitals. Each sample was divided into three parts, one of which was stored in cold storage and the other two were refrigerated and transported to the other two laboratories. After receiving the samples, the three laboratories conducted testing on the same day and then compared and analyzed the results to understand the consistency of the results. If there was a significant difference, the results should be consistent according to mathematical calculation prior to further analysis.

### Statistical analysis

SPSS 22.0 software was used to conduct the statistics. A repeated measurement analysis of variance was used to understand the consistency of the RBT results from the three laboratories. A two-factor analysis of variance (ANOVA) was used to understand the age differences between the different regions and ethnic groups. A two-factor ANOVA with one covariate was used to understand the influence of region, ethnicity, and age on the results. A Chi-square test was used to understand the difference in an abnormal proportion of the results between the different regions and ethnic groups. *P* < 0.05 was considered to be statistically significant.

## Results

### Minimum sample size

According to the calculation results (Tables 1, 2) combined with the actual situation, this study included 100 cases in each of six groups: the Sanya Li nationality (SL), the Sanya Han nationality (SH), the Xining Han nationality (XH), the Xining Tibetan nationality (XZ), the Maduo Han nationality (MH), and the Maduo Tibetan nationality (MZ) for a total of 600 cases.

### Consistency of the routine blood test results

The results of the repeated measurement analysis of the variance showed that there was no significant difference in the values of the RBC (*P* = 0.801), HB (*P* = 0.393), HCT (*P* = 0.852), MCV (*P* = 0.580), MCH (*P* = 0.420), MCHC (*P* = 0.085), PCT (*P* = 0.116), and PLT (*P* = 0.910). The above results suggested that the RBT results of the three laboratories were consistent, and the data could be further analyzed.

### Age distribution

A total of 643 subjects were enrolled in this study, of which 16 cases were excluded from the clinical diagnoses of hepatitis, nephritis, thalassemia, leukemia, severe infection, and other diseases. A total of 617 cases were actually included in the study, with an average age of 33.5 ± 6.0 years. The age distribution of the enrolled population is shown in Table 3. The results of the two-way ANOVA showed that there were significant differences in age among the research objects (*P* = 0.003). Therefore, in the subsequent analysis of variance of the RBT, the “age” factor was included as a covariate.

**Table 3:** Age distribution of the enrolled population ($\bar{x}\pm s$, *n*).

**Ethnic**	**Region**			**Total**
	**Sanya (200 m)**	**Xining (2,300 m)**	**Maduo (4,300 m)**	
Li	35.2 ± 7.5, 94	–	–	35.2 ± 7.5, 94
Han	33.7 ± 6.2, 96	31.9 ± 4.7, 102	33.6 ± 6.5, 94	33.0 ± 5.9, 292
Tibetan	–	34.1 ± 5.7, 109	32.7 ± 5.1, 122	33.3 ± 5.5, 231
Total	34.5 ± 6.9, 190	33.0 ± 5.4, 211	33.1 ± 5.8, 216	33.5 ±6.0, 617

*$\bar{x}$, mean value; s, standard deviation*.

### RBT results

**Figure 1:**
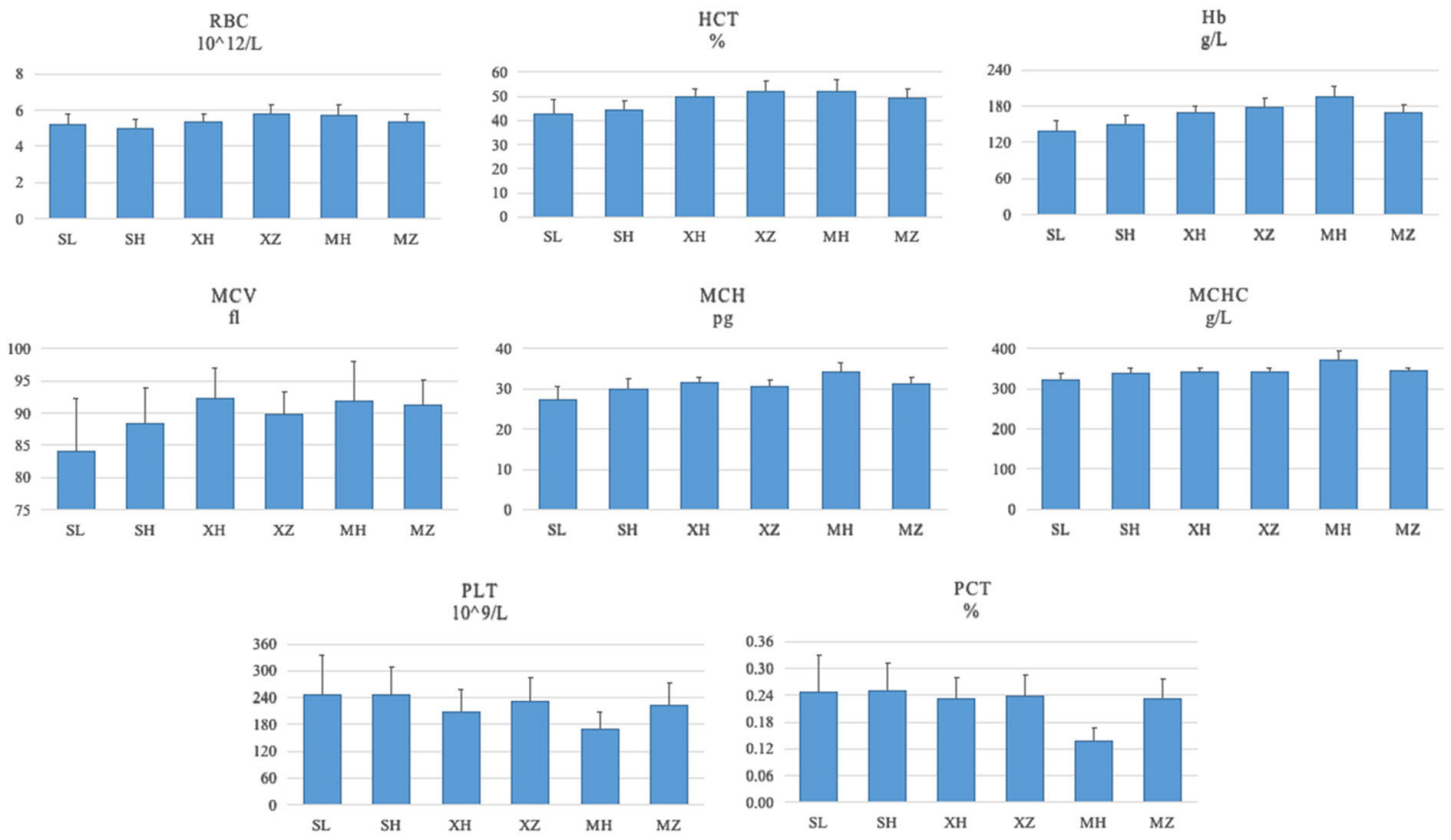
RBT results of the enrolled population ($\bar{x}\pm s$). SL, the Sanya Li nationality; SH, the Sanya Han nationality; XH, the Xining Han nationality; XZ, the Xining Tibetan nationality; MH, the Maduo Han nationality; MZ, and the Maduo Tibetan nationality. RBC, red blood cell count; HCT, hematocrit; HB, hemoglobin content; MCV, mean red blood cell volume; MCH, mean red blood cell hemoglobin; MCHC, mean red blood cell hemoglobin concentration; PLT, platelet count; PCT, platelet specific volume.

The RBT results of the enrolled population are shown in Table 4 and Figure 1. The populations with the lowest/highest mean values of each index were RBC SH/XZ (5.02/5.81 10^∧^12/L), HCT SL/XZ (42.7/52.0%), Hb SL/MH (138/195 g/L), MCV SL/XH (84.0/92.3 fl), MCH SL/MH (27.2/34.1 pg), MCHC SL/MH (323/371 g/L), PLT MH/SL (169/246 10^∧^9/L), and PCT MH/SH (0.138/0.251%).

**Table 4:** RBT results of the enrolled population ($\bar{x}\pm s$).

**Index**	**Ethnic**	**Region**				**Significance** **(*****P***=**)**	
		**Sanya**	**Xining**	**Maduo**	**Total**	
RBC (10^∧^12/L)	Li	5.19 ± 0.62	–	–	5.19 ± 0.62	Total	0.000
	Han	5.02 ± 0.50	5.35 ± 0.42	5.70 ± 0.58	5.35 ± 0.57	Region	0.000
	Tibetan	–	5.81 ± 0.52	5.38 ± 0.43	5.58 ± 0.52	Ethnic	0.032
	Total	5.11 ± 0.57	5.58 ± 0.53	5.52 ± 0.52	5.42 ± 0.58	Region*Ethnic	0.000
HCT (%)	Li	42.7 ± 6.2	–	–	42.7 ± 6.2	Total	0.000
	Han	44.3 ± 4.1	49.6 ± 3.6	51.7 ± 5.0	48.6 ± 5.2	Region	0.000
	Tibetan	–	52.0 ± 4.1	49.1 ± 4.2	50.5 ± 4.4	Ethnic	0.032
	Total	43.5 ± 5.3	50.9 ± 4.0	50.2 ± 4.7	48.4 ± 5.7	Region*Ethnic	0.000
Hb (g/L)	Li	138 ± 17	–	–	138 ± 17	Total	0.000
	Han	150 ± 15	168 ± 12	195 ± 19	171 ± 24	Region	0.000
	Tibetan	–	178 ± 15	169 ± 14	173 ± 15	Ethnic	0.000
	Total	144 ± 17	173 ± 14	180 ± 21	167 ± 23	Region*Ethnic	0.000
MCV (fl)	Li	84.0 ± 8.2	–	–	84.0 ± 8.2	Total	0.000
	Han	88.4 ± 5.5	92.3 ± 4.8	91.8 ± 6.1	90.9 ± 5.7	Region	0.000
	Tibetan	–	89.8 ± 3.6	91.2 ± 4.0	90.6 ± 3.8	Ethnic	0.000
	Total	86.2 ± 7.3	91.0 ± 4.4	91.5 ± 5.0	89.7 ± 6.1	Region*Ethnic	0.071
MCH (pg)	Li	27.2 ± 3.5	–	–	27.2 ± 3.5	Total	0.000
	Han	30.0 ± 2.5	31.4 ± 1.6	34.1 ± 2.3	31.8 ± 2.8	Region	0.000
	Tibetan	–	30.7 ± 1.5	31.4 ± 1.3	31.1 ± 1.4	Ethnic	0.000
	Total	28.6 ± 3.3	31.0 ± 1.6	32.6 ± 2.3	30.8 ± 3.0	Region*Ethnic	0.000
MCHC (g/L)	Li	323 ± 16	–	–	323 ± 16	Total	0.000
	Han	338 ± 14	341 ± 11	371 ± 22	350 ± 22	Region	0.000
	Tibetan	–	342 ± 9	343 ± 8	343 ± 8	Ethnic	0.000
	Total	331 ± 17	341 ± 10	355 ± 21	343 ± 19	Region*Ethnic	0.000
PLT (10^∧^9/L)	Li	246 ± 89	–	–	246 ± 89	Total	0.000
	Han	245 ± 64	208 ± 49	169 ± 38	208 ± 60	Region	0.000
	Tibetan	–	230 ± 54	223 ± 50	227 ± 52	Ethnic	0.000
	Total	245 ± 77	219 ± 53	200 ± 52	221 ± 64	Region*Ethnic	0.007
PCT (%)	Li	0.246 ± 0.084	–	–	0.246 ± 0.084	Total	0.000
	Han	0.251 ± 0.059	0.232 ± 0.047	0.138 ± 0.028	0.208 ± 0.067	Region	0.000
	Tibetan	–	0.238 ± 0.047	0.233 ± 0.043	0.235 ± 0.045	Ethnic	0.000
	Total	0.248 ± 0.072	0.235 ± 0.047	0.192 ± 0.060	0.224 ± 0.065	Region*Ethnic	0.000

*RBC, red blood cell count; HCT, hematocrit; HB, hemoglobin content; MCV, mean red blood cell volume; MCH, mean red blood cell hemoglobin; MCHC, mean red blood cell hemoglobin concentration; PLT, platelet count; PCT, platelet specific volume*.

Table 4 shows the results of the two-way ANOVA with “age” as a covariate that there were significant differences in RBC, HCT, Hb, MCV, MCH, MCHC, PCT, and PLT among the 617 enrolled people (*P* = 0.000). Among them, “region” (*P* = 0.000) and “nationality” (RBC, HCT, *P* = 0.032, and the rest *P* = 0.000) had significant effects on these indexes. In addition, with the exception of MCV (*P* = 0.071), “region” and “nationality” had significant interactive effects with the other seven indicators (PLT *P* = 0.007, the rest *P* = 0.000). The results suggested that regional factors had different effects on the RBT results of the different ethnic groups.

### Abnormal proportion RBT results

**Figure 2:**
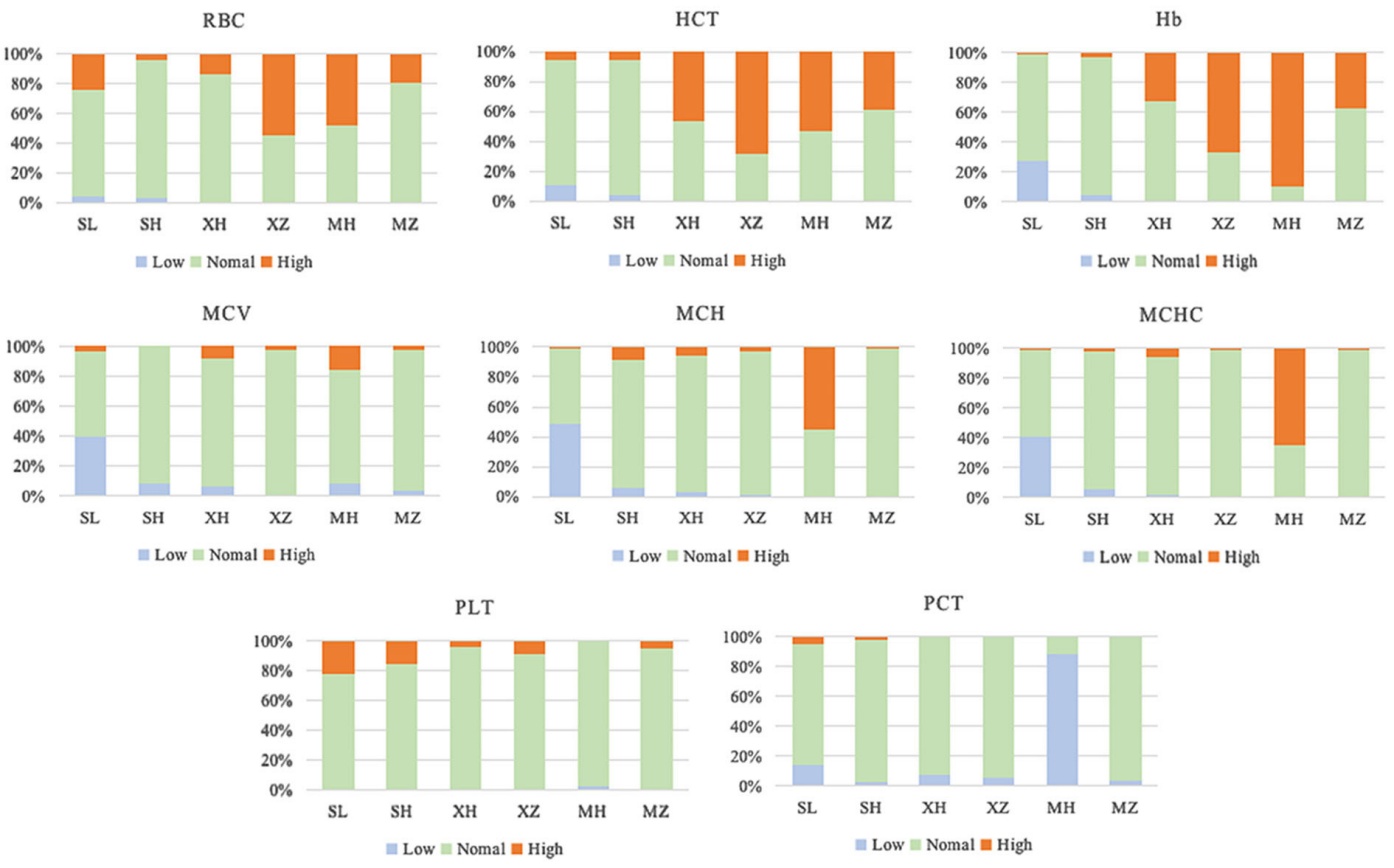
RBT abnormal rate of the enrolled population. SL, the Sanya Li nationality; SH, the Sanya Han nationality; XH, the Xining Han nationality; XZ, the Xining Tibetan nationality; MH, the Maduo Han nationality; MZ, and the Maduo Tibetan nationality. RBC, Red blood cell count; HCT, hematocrit; HB, hemoglobin content; MCV, mean red blood cell volume; MCH, mean red blood cell hemoglobin; MCHC, mean red blood cell hemoglobin concentration; PLT, platelet count; PCT, platelet specific volume.

The current reference values of the RBT for adult men in China are as follows (4): RBC 4.09–5.74 10^∧^12/L, HCT 35–50%, Hb 131–172 g/L, MCV 83.9–99.1 fl, MCH 27.8–33.8 pg, MCHC 320–360 g/L, PLT 100–300 10^∧^9/L, and PCT 0.17–0.38%. According to the above reference value ranges, the abnormal RBT rate of the enrolled population was calculated and evaluated. The results showed that (Table 5; Figure 2), when referring to the current reference value range, the abnormal rates of RBT in the different regions and ethnic groups were different. Among them, the RBC of XZ (55.0%), HCT of XZ (67.9%)/MH (53.2%), Hb of XZ (67.0%)/MH (89.4%)/SL (50.0%), MCH of MH (55.3%), MCHC of MH (64.9%), and PCT of MH (88.3%) all had abnormal rates of >50%, and the highest one was 89.4%.

**Table 5:** RBT abnormal rate of the enrolled population (%).

**Index**	**Ethnic**	**Region**																**Significance (*****P***=**)**
		**Sanya**				**Xining**				**Maduo**				**Total**				
		***Low***	***High***	***Add***	***n***	***Low***	***High***	***Add***	***n***	***Low***	***High***	***Add***	***n***	***Low***	***High***	***Add***	***n***	
RBC	Li	4.3%	24.5%	28.7%	94	–	–	–	–	–	–	–	–	4.3%	24.5%	28.7%	94	Region 0.927 Ethnic 0.000
	Han	3.1%	4.2%	7.3%	96	0.0%	13.7%	13.7%	102	0.0%	47.9%	47.9%	94	1.0%	21.6%	22.6%	292	
	Tibetan	–	–	–	–	0.0%	55.0%	55.0%	109	0.0%	19.7%	55.0%	122	0.0%	36.4%	36.4%	231	
	Total	3.7%	14.2%	17.9%	190	0.0%	35.1%	35.1%	211	0.0%	31.9%	35.1%	216	1.1%	27.6%	28.7%	617	
HCT	Li	10.6%	5.3%	16.0%	94	–	–	–	–	–	–	–	–	10.6%	5.3%	16.0%	94	Region 0.267 Ethnic 0.000
	Han	4.2%	5.2%	9.4%	96	0.0%	46.1%	46.1%	102	0.0%	53.2%	46.1%	94	1.4%	34.9%	36.3%	292	
	Tibetan	–	–	–	–	0.0%	67.9%	67.9%	109	0.0%	38.5%	67.9%	122	0.0%	52.4%	52.4%	231	
	Total	7.4%	5.3%	12.6%	190	0.0%	57.3%	57.3%	211	0.0%	44.9%	57.3%	216	2.3%	37.0%	39.2%	617	
Hb	Li	27.7%	1.1%	28.7%	94	–	–	–	–	–	–	–	–	27.7%	1.1%	28.7%	94	Region 0.000 Ethnic 0.000
	Han	4.2%	3.1%	7.3%	96	0.0%	32.4%	32.4%	102	0.0%	89.4%	32.4%	94	1.4%	41.1%	42.5%	292	
	Tibetan	–	–	–	–	0.0%	67.0%	67.0%	109	0.8%	36.9%	67.0%	122	0.4%	51.1%	51.5%	231	
	Total	15.8%	2.1%	17.9%	190	0.0%	50.2%	50.2%	211	0.5%	59.7%	50.2%	216	5.0%	38.7%	43.8%	617	
MCV	Li	39.4%	3.2%	42.6%	94	–	–	–	–	–	–	–	–	39.4%	3.2%	42.6%	94	Region 0.000 Ethnic 0.003
	Han	8.3%	0.0%	8.3%	96	5.9%	7.8%	13.7%	102	8.5%	16.0%	13.7%	94	7.5%	7.9%	15.4%	292	
	Tibetan	–	–	–	–	0.9%	2.8%	3.7%	109	3.3%	2.5%	3.7%	122	2.2%	2.6%	4.8%	231	
	Total	23.7%	1.6%	25.3%	190	3.3%	5.2%	8.5%	211	5.6%	8.3%	8.5%	216	10.4%	5.2%	15.6%	617	
MCH	Li	48.9%	1.1%	50.0%	94	–	–	–	–	–	–	–	–	48.9%	1.1%	50.0%	94	Region 0.000 Ethnic 0.386
	Han	5.7%	8.6%	14.3%	105	2.9%	5.9%	8.8%	102	0.0%	55.3%	8.8%	94	3.0%	22.3%	25.2%	301	
	Tibetan	–	–	–	–	0.9%	2.8%	3.7%	109	0.0%	0.8%	3.7%	122	0.4%	1.7%	2.2%	231	
	Total	26.1%	5.0%	31.2%	199	1.9%	4.3%	6.2%	211	0.0%	24.5%	6.2%	216	8.9%	11.5%	20.4%	626	
MCHC	Li	40.4%	1.1%	41.5%	94	–	–	–	–	–	–	–	–	40.4%	1.1%	41.5%	94	Region 0.000 Ethnic 0.385
	Han	5.2%	2.1%	7.3%	96	2.0%	5.9%	7.8%	102	0.0%	64.9%	7.8%	94	2.4%	23.6%	26.0%	292	
	Tibetan	–	–	–	–	0.0%	0.9%	0.9%	109	0.0%	0.8%	0.9%	122	0.0%	0.9%	0.9%	231	
	Total	22.6%	1.6%	24.2%	190	0.9%	3.3%	4.3%	211	0.0%	28.7%	4.3%	216	7.3%	11.7%	19.0%	617	
PLT	Li	0.0%	22.3%	22.3%	94	–	–	–	–	–	–	–	–	0.0%	22.3%	22.3%	94	Region 0.165 Ethnic 0.858
	Han	0.0%	15.6%	15.6%	96	0.0%	3.9%	3.9%	102	2.1%	0.0%	3.9%	94	0.7%	6.5%	7.2%	292	
	Tibetan	–	–	–	–	0.0%	9.2%	9.2%	109	0.0%	4.9%	9.2%	122	0.0%	6.9%	6.9%	231	
	Total	0.0%	18.9%	18.9%	190	0.0%	6.6%	6.6%	211	0.9%	2.8%	6.6%	216	0.3%	9.1%	9.4%	617	
PCT	Li	13.8%	5.3%	19.1%	94	–	–	–	–	–	–	–	–	13.8%	5.3%	19.1%	94	Region 0.466 Ethnic 0.000
	Han	2.1%	2.1%	4.2%	96	6.9%	0.0%	6.9%	102	88.3%	0.0%	6.9%	94	31.5%	0.7%	32.2%	292	
	Tibetan	–	–	–	–	5.5%	0.0%	5.5%	109	3.3%	0.0%	5.5%	122	4.3%	0.0%	4.3%	231	
	Total	7.9%	3.7%	11.6%	190	6.2%	0.0%	6.2%	211	40.3%	0.0%	6.2%	216	18.6%	1.1%	19.8%	617	

*RBC, Red blood cell count; HCT, hematocrit; HB, hemoglobin content; MCV, mean red blood cell volume; MCH, mean red blood cell hemoglobin; MCHC, mean red blood cell hemoglobin concentration; PLT, platelet count; PCT, platelet specific volume*.

## Discussion

### Regarding the selection of the research objects

This study was a retrospective cross-sectional study that aimed to further explore the applicability of the current RBT reference range to different populations by understanding the influence of “region” and “nationality” on the RBT of healthy men.

The Li nationality is a minority that has been living on Hainan Island for generations, with a total population of approximately 1.6 million (5). In addition, the average altitude of residence is only 3–120 m (6). Tibetans have lived in Qinghai, Tibet, and other plateau areas for generations, with an average altitude of approximately 4,000 m and a total population of approximately 4.6 million (7). Although studies have reported physiological differences between the Li/Han population and the Tibetan/Han population, the settlements of the Li and Tibetan nationalities are far apart, and previous studies involving the two ethnic groups at the same time have rarely been reported. In addition, because it is difficult to access the Tibetan people who have lived in Sanya or the Li people who have lived on the plateau for a long time, we included the Li, Han, and Tibetan people who live in Sanya, Xining, and Maduo that have altitude differences of 2,000 m.

### Regional factors have a significant impact on RBT results

We found that the RBC, HCT, Hb, MCV, MCH, and MCHC increased by 6.6, 8.1, 14.0, 1.9, 6.7, and 4.8%, respectively, for every 2,000 m increase in altitude of the long-term residence of young Han men. The results suggested that with an increase in altitude, the number, volume, and hemoglobin of red blood cells in the Han population increased, which was consistent with many research reports (2, 3, 8–11). This is because with an increase in the altitude of the residence, to adapt to the hypoxic environment, the body's erythropoietin increases and the production of bone marrow erythrocytes is active, resulting in physiological compensatory changes in the erythrocytes.

According to the research report (12, 13), a short-term high altitude hypoxia environment can lead to an increase in the PLT, but a decrease in the PLT is more common in a long-term hypoxia environment. Our results showed that the PLT and PCT values of the Han population decreased by 16.8 and 24.0%, respectively, for every 2,000 m increase in altitude. Some studies have pointed out that (14, 15) in a long-term hypoxic environment, thrombocytopenia can reduce the increase in the blood viscosity caused by an increase in erythrocytes to some extent, and this is conducive to microcirculation perfusion so as to increase tissue oxygen uptake. In addition, hypoxia can inhibit the differentiation of hematopoietic stem cells (HSCs) into megakaryocytes (MKS), reduce the number of mature MKS, and thus reduce platelet production (12). The physiological and cellular of these changes in erythrocytes and platelets in people at high altitude requires further investigation.

### Ethnic factors had a significant impact on the RBT results

We found that even if living in the same region, there were still significant differences in the RBT among the different ethnic groups. Among them, the RBC (*P* = 0.036) of the Li nationality in Sanya was significantly higher than that of the Han nationality. However, the values of the HCT (*P* = 0.030), HB (*P* = 0.000), MCV (*P* = 0.000), MCH (*P* = 0.000), and MCHC (*P* = 0.000) were significantly lower than that of the Han nationality. In addition, the RBC, HCT, HB (*P* = 0.000), and PLT (*P* = 0.002) of the Tibetan population in the Xining area were significantly higher than those of the Han nationality, but the MCV (*P* = 0.000) and MCH (*P* = 0.001) were significantly lower than those of the Han nationality (*P* = 0.000). This result was basically consistent with the literature (16). In addition, the RBC, HCT, Hb, MCH, and MCHC of the Tibetan population in the Maduo area were significantly lower than those of the Han nationality, but the PLT and PCT were significantly higher (*P* = 0.000).

In addition, we also found that when the altitude changed, there were differences in the changing trend of blood routine indicators among the different ethnic groups. For example, when the residence increased by 2,000 m (Xining/Maduo), the Han population showed increased RBC (5.35/5.70 10^∧^12/L), Hb (168/195 g/L), and PLT (208/169 10^∧^9/L). However, the Tibetan population showed a decrease in the RBC (5.81/5.38 10^∧^12/L), Hb (178/169 g/L), and PLT (230/223 10^∧^9/L). The specific reasons and mechanisms leading to this difference require further investigation.

### Current RBT reference value range needs to be revised

RBT is one of the most commonly used test items to evaluate the human physiological state in the clinic. The establishment of its reference value is of great significance to the accuracy of clinical diagnosis. China is a vast territory with great differences in regional altitudes and ethnic compositions of the population. However, the current RBT reference values are divided only according to age and gender but do not include regional and ethnic divisions.

We found that the abnormal rate of healthy Han people in low altitude areas (200 m) was only 4.2–15.6%. However, at high altitudes (2,000–4,000 m), the abnormal rate even exceeded 1/3, such as RBC > 31%, HCT > 44%, and Hb > 50%. Similar phenomena have been reported in many studies (2, 8, 10, 17, 18). Hence, some scholars have proposed that the reference value range of the RBT should be revised according to regional differences. Based on a large-scale data analysis, Zhu et al. (17) and Fu et al. (19) deduced a revision equation of the Hb reference value that substituted the regional altitude, annual sunshine duration, annual average relative humidity, annual average temperature, annual precipitation, and other data into the equation. They then calculated that the reference value range for Hb for 18–25-year-old men in Qinghai was 147–193 g/L (17, 19), and for 26–45 years old it was 149–185 g/L (17). Based on the revised reference value range, the Hb value of 195 men (26–45 years old, including Han and Tibetan) in the Xining area in this study was evaluated again, and the abnormal rate was 20.0%, which was far lower than the abnormal rate (50.3%) evaluated based on the current reference value range (131–172 g/L).

In addition, we found that the abnormal rate of ethnic minorities was also high when we evaluated using the current reference value range. Among the 231 Tibetans included in this study, the high values of the RBC, HCT, and Hb accounted for 36.4, 52.4, and 51.1%, respectively. Among the 94 Li people, 24.5% RBC and 22.3% PLT were high, while 27.7% Hb, 39.4% MCV, 48.9% MCV, and 40.4% MCHC were low. Zhao et al. (20) reported that among the Buyi, Miao, Shui, and Yao nationalities (*n* = 369) living in Libo County, Guizhou Province, a high Hb accounted for 37.67%. It can be seen that the current blood routine reference value range is not accurate for ethnic minorities.

Therefore, we believe that the current RBT reference value range is not applicable to people in high-altitude areas and ethnic minorities, and it urgently requires adjustment.

### Some ideas regarding the RBT characteristics of the li nationality

In 2004 (21) and 2009 (22), studies reported that the incidence of anemia in Li men was 60.0% (21), and even as high as 69.2% in the poor mountainous areas (22). Compared with the corrected Hb reference value range, 47.2% of Hainan residents were still anemic, which is much higher than the average level of 20.1% in China at that time (21). In this study, they believed that economic backwardness was the primary reason for the high incidence of anemia among the Li people (22). Studies in 2016 (16) and 2017 (23) found that the incidence of anemia among the Li people on Hainan Island was significantly higher than that of the Han people (Han men: 3.1–3.7%, Li men: 7.1–8.0%, Han women: 2.5–8.0%, Li women: 11.3–17.1%). In this study, we found that 27.7% of the Li men in Sanya met the World Health Organization diagnostic criteria for anemia (Hb <130 g/L), but this proportion was only 8.3% in the Han men in Sanya. It can be seen that the Li population has unique hematological index characteristics.

A 2020 study found that the content of hair mercury increased significantly with fish consumption in adolescents living on Tira Bamba Island on the Caribbean coast of Colombia (24). Studies have confirmed that the content of hair mercury is negatively correlated with the content of Hb in the population (25). A study on the content of trace elements in 114 aquatic products from 8 coastal cities in China found that fish were the primary contributor to human mercury intake (26). As the Li people have lived on Hainan Island for generations, they eat a typical Mediterranean diet, and marine fish occupy an important position in their diet (27). Therefore, we speculate that the low Hb content of the Li people may be caused by the increase in blood mercury content due to the large intake of marine fish.

In addition, a large number of studies have confirmed that the Tibetan people have an increased adaptability to Hb and decreased adaptability to PLT due to living in a hypoxic environment for generations. This phenomenon is considered to be the result of long-term acclimatization of the human body to a plateau environment (3, 28). We found that there were changes in the number, volume, and hemoglobin content of red blood cells in the Li population. Thalassemia is caused by Hb α or β autosomal recessive genetic diseases caused by reduced chain synthesis, and this is common in tropical countries (29). In 1982, a survey report on thalassemia based on 100,000 people in Hainan Province showed that the incidence of HbH disease in the Li population was 0.233%, much higher than the 0.027% in the Han population (30). Since then, studies have reported that in the Li population in Hainan, the α-thalassemia gene carrying rate is 53.9–57.2%, while that of the Han nationality is 4.5–21.56% (31–33). It is considered that the low Hb in the Li population may be related to the presence of the thalassemia gene (16).

The subjects in this study were all healthy adult Li men, but there was still a high abnormal rate of RBT. Therefore, we believe that the high incidence of anemia and the high carrying rate of lean genes in the Li population is the result of the acclimatization of the human body to the extremely low altitude and high oxygen environment, which is a change of physiological adaptation and is not pathological.

## Conclusion

This study analyzed the RBT results of 617 healthy men aged 18–45 years old of the Li, Tibet, and Han nationalities who have lived in Sanya, Xining, and Maduo. We found that regional and ethnic factors had significant effects on eight RBT indexes, such as the RBC, Hb, and PLT. With the current reference value range, it is impossible to completely and accurately evaluate whether the blood routine indicators of special populations, such as high-altitude areas and ethnic minorities, are normal. This is very unfavorable to the diagnosis and prevention of diseases in such populations. Therefore, it is urgent to incorporate regional and ethnic factors to revise and adjust the current normal reference value range of the RBT.

## Data availability statement

The raw data supporting the conclusions of this article will be made available by the authors, without undue reservation.

## Ethics statement

The studies involving human participants were reviewed and approved by Ethics Committee of Qinghai Provincial People's Hospital (2021-41) and Hainan Hospital of PLA General Hospital. Written informed consent for participation was not required for this study in accordance with the national legislation and the institutional requirements.

## Author contributions

Data collections were performed by YG, ZZ, NZ, and ZS. Data analysis was performed by XL. The first draft of the manuscript was written by XL and QZ. All authors contributed to the study conception and design, commented on previous versions of the manuscript, and approved the final manuscript.

## Funding

This work was supported by the Applied basic research project of Qinghai basic research plan, No. 2022-zj-765 and the Guiding scientific research project of health in Qinghai Province, No. 2021-wjzsx.

## Conflict of interest

The authors declare that the research was conducted in the absence of any commercial or financial relationships that could be construed as a potential conflict of interest.

## Publisher's note

All claims expressed in this article are solely those of the authors and do not necessarily represent those of their affiliated organizations, or those of the publisher, the editors and the reviewers. Any product that may be evaluated in this article, or claim that may be made by its manufacturer, is not guaranteed or endorsed by the publisher.
